# 2155. Low prevalence of OXA-48-like carbapenemases and susceptibility to imipenem/relebactam among carbapenem-resistant non-*Morganellaceae* Enterobacterales isolates from Latin America – SMART 2018-2021

**DOI:** 10.1093/ofid/ofad500.1778

**Published:** 2023-11-27

**Authors:** Sibylle Lob, Mark G Wise, Fakhar Siddiqui, Thales Polis, C Andrew DeRyke, Katherine Young, Mary Motyl, Daniel F Sahm

**Affiliations:** Merck & Co., Inc., Schaumburg, Illinois; IHMA, Schaumburg, Illinois; Merck & Co., Inc., Schaumburg, Illinois; MSD Brazil, Sao Paulo, Brazil, Sao Paulo, Sao Paulo, Brazil; Merck & Co., Inc., Schaumburg, Illinois; Merck, Rahway, New Jersey; Merck, Rahway, New Jersey; IHMA, Schaumburg, Illinois

## Abstract

**Background:**

Imipenem/relebactam (IMI/REL) is a combination of imipenem/cilastatin with relebactam, an inhibitor of class A and C β-lactamases. IMI/REL is not active against metallo-β-lactamases (MBLs) or OXA-48-like carbapenemases. Using non-*Morganellaceae* Enterobacterales (NME) isolates collected in 9 countries in Latin America as part of the SMART surveillance program, we evaluated the proportion of carbapenemases among carbapenem-resistant (CR) isolates as well as the activity of IMI/REL against all and CR isolates.

**Methods:**

In 2018-2021, 44 clinical labs in Argentina, Brazil, Chile, Colombia, Ecuador, Guatemala, Mexico, Panama, and Puerto Rico each collected up to 250 consecutive, gram-negative pathogens per year from patients with bloodstream, intraabdominal, respiratory tract, and urinary tract infections. MICs were determined using CLSI broth microdilution and interpreted with CLSI breakpoints. Isolates that were imipenem-, IMI/REL-, or ceftolozane/tazobactam-nonsusceptible were screened for β-lactamases. Carbapenem-resistance was defined as nonsusceptibility (I+R) to meropenem.

**Results:**

Susceptibility to IMI/REL among all collected NME isolates from Latin America was 96.5%, ranging from 90% (Guatemala) to 99% (Puerto Rico) (Figure 1). Among CR isolates, IMI/REL-susceptibility was 72.0% overall, ranging from 4% (Guatemala) to 95% (Puerto Rico). Carbapenem-resistance among NME isolates collected in Latin America was mostly attributable to carriage of MBLs or KPCs, while OXA-48-like and GES carbapenemases were rare (Figure 2). Lower IMI/REL-susceptibility of CR NME from Guatemala, Mexico, and Panama correlated with higher MBL rates among CR NME (37-93%), while IMI/REL activity against CR NME in countries with high KPC rates (≥80% in Argentina, Brazil, Colombia, Ecuador, and Puerto Rico) remained high (73-95% susceptible). Rates of isolates carrying OXA-48-like carbapenemases among CR NME were low everywhere (≤2% in all countries except Mexico [14%]).

**Figure:**
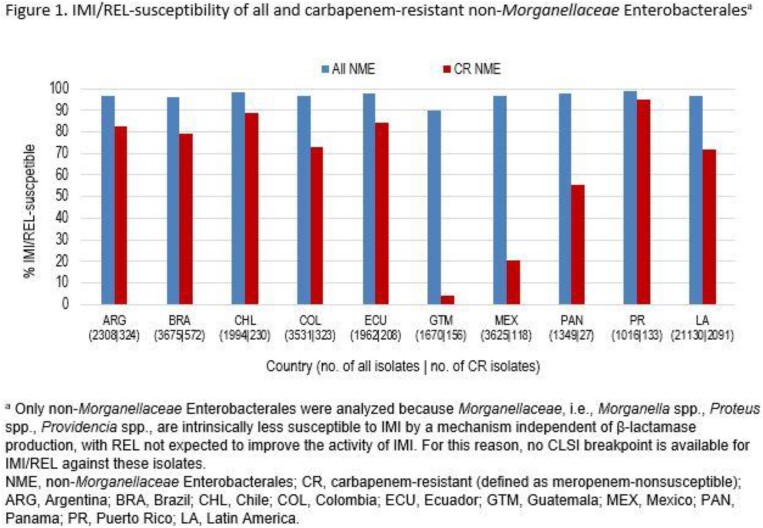

**Figure:**
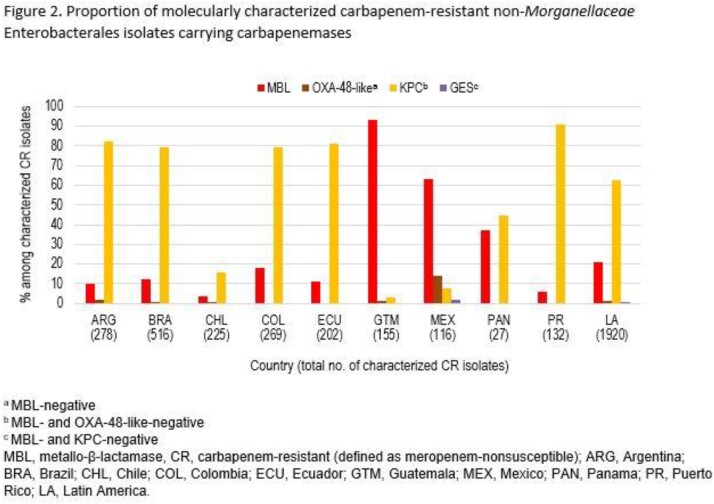

**Conclusion:**

IMI/REL showed strong activity against carbapenem-resistant NME isolates collected in most countries in Latin America. Susceptibility was only reduced in countries with higher MBL rates, while OXA-48-like carbapenemases were detected rarely.

**Disclosures:**

**Sibylle Lob, MD**, Merck & Co., Inc.: Honoraria **Mark G Wise, PhD**, Merck & Co., Inc.: Honoraria|Pfizer Inc.: Honoraria|Venatorx: Paid fees for conducting the study and abstract preparation **Fakhar Siddiqui, MD, MBA**, Merck & Co Inc.: Employee **Daniel F. Sahm, PhD**, Merck & Co., Inc.: Honoraria|Pfizer Inc.: Honoraria|Venatorx: Paid fees for conducting the study and abstract preparation

